# Evaluating the discriminatory power of the velocity field diagram and timed-up-and-go test in determining the fall status of community-dwelling older adults: a cross-sectional observational study

**DOI:** 10.1186/s12877-022-03282-2

**Published:** 2022-08-11

**Authors:** Sam Chidi Ibeneme, Joy Chinyere Eze, Uchenna Prosper Okonkwo, Georgian Chiaka Ibeneme, Gerhard Fortwengel

**Affiliations:** 1 Department of Medical Rehabilitation, Faculty of Health Sciences, University of Nigeria, Enugu Campus, Enugu, Enugu State Nigeria; 2 Department of Physiotherapy, Nnamdi Azikiwe University Teaching Hospital, Nnewi, Nigeria; 3 Department of Nursing Sciences, Ebonyi State University Teaching Hospital, Abakaliki, Ebonyi State Nigeria; 4 Faculty III/Mid–Research Group, Hochschule Hannover - University of Applied Sciences and Arts, Hannover, Expo Plaza 12, 30539 Hannover, Germany; 5 Department of Physiotherapy, Faculty of Health Sciences, School of Therapeutic Studies, University of the Witwatersrand, 7 York Road, Parktown, Johannesburg, 2193 South Africa; 6 King David University of Medical Sciences, Ubulu, Ebonyi State Nigeria; 7 Clinical Trials Consortium, University of Nigeria, Nsukka, Nigeria

**Keywords:** Community-dwelling older adults, Falls, Velocity field diagram, Timed-up-and-go test, Discriminatory power, Validity, Reliability

## Abstract

**Background:**

Systematic reviews demonstrated that gait variables are the most reliable predictors of future falls, yet are rarely included in fall screening tools. Thus, most tools have higher specificity than sensitivity, hence may be misleading/detrimental to care. Therefore, this study aimed to determine the validity, and reliability of the velocity field diagram (VFD -a gait analytical tool), and the Timed-up-and-go test (TUG)-commonly used in Nigeria as fall screening tools, compared to a gold standard (known fallers) among community-dwelling older adults.

**Method:**

This is a cross-sectional observational study of 500 older adults (280 fallers and 220 non-fallers), recruited by convenience sampling technique at community health fora on fall prevention. Participants completed a 7-m distance with the number of steps and time it took determined and used to compute the stride length, stride frequency, and velocity, which regression lines formed the VFD. TUG test was simultaneously conducted to discriminate fallers from non-fallers. The cut-off points for falls were: TUG times ≥ 13.5 s; VFD’s intersection point of the stride frequency, and velocity regression lines (E_1_) ≥ 3.5velots. The receiver operating characteristic (ROC) area under the curves (AUC) was used to explore the ability of the E_1_ ≥ 3.5velots to discriminate between fallers and non-fallers. The VFD’s and TUG’s sensitivity, specificity, positive predictive value (PPV), and negative predictive value (NPV) were determined. Alpha was set at *p* < 0.05.

**Results:**

The VFD versus TUG sensitivity, specificity, PPV and NPV were 71%, 27%, 55%, and 42%, versus 39%, 59%, 55%, and 43%, respectively. The ROC’s AUC were 0.74(95%CI:0.597,0.882, p = 0.001) for the VFD. The optimal categorizations for discrimination between fallers/non-fallers were ≥ 3.78 versus ≤ 3.78 for VFD (fallers versus non-fallers prevalence is 60.71% versus 95.45%, respectively), with a classification accuracy or prediction rate of 0.76 unlike TUG with AUC = 0.53 (95% CI:0.353,0.700, *p* = 0.762), and a classification accuracy of 0.68, and optimal characterization of ≥ 12.81 s versus ≤ 12.81 (fallers and non-fallers prevalence = 92.86% versus 36.36%, respectively).

**Conclusion:**

The VFD demonstrated a fair discriminatory power and greater reliability in identifying fallers than the TUG, and therefore, could replace the TUG as a primary tool in screening those at risk of falls.

## Introduction

Falls occur when an individual unintentionally rests in a new position on the ground and is therefore related to the instability of the body’s centre of gravity in an upright stance. Falls account for 40% of all injury deaths as well as 20–30% of mild to severe injuries (including soft tissue injuries to fractures) in older adults [1–5]. This warrants the development of fall prevention strategies which include screening those at the risk of falls and targeting them with appropriate fall prevention measures before a fall occurs. Some simple and practical fall screening methods are advocated [6–9], including the Timed-Up-and-Go test (TUG) [9] and velocity field diagram (VFD) [10, 11] as cumbersome and highly sophisticated equipment are not desirable considering the difficulty in deploying them in various settings [12]. However, several systematic reviews found none of the 29 fall-risk assessment tools (including the TUG) accurate enough to identify people at risk of falling in hospitals and care homes [13–15]. For instance, some screening tests had higher specificity than sensitivity [15], indicating that a higher proportion of non-fallers than fallers were correctly identified. Perhaps, this limitation relates to the non-inclusion of the best predictors of falls (i.e. history of falls, gait abnormalities, and balance assessment) in some screening tools [13, 14]. Given that the VFD detects gait abnormalities, balance dysfunctions, and fallers [16–18], it may be a more reliable instrument than the commonly used TUG which shows some weaknesses in this area.

Some fall screening tools include the Get-up-and-Go test [19], the TUG test, the Bohannon’s ordinal scale for standing balance [20], Berg balance tests [6], Bartel Index for activities of daily life [21], Tinetti’s sub-scale for balance [22], Fugl-Meyer scale for isolated movements and balance [23],Minimal Chair Height Standing Ability Test (MCHSAT) [24] and a test of functional mobility [25]. But unlike the VFD, some of the above instruments focus on the assessment of motor performances in non-ambulant activities of daily living (ADL), while others (e.g. TUG) employ gait assessment at ordinary walking speed alone, over a distance of 3 m. This is an obvious weakness, which does not allow for a full expression of gait pattern across the entire spectrum of (slow, normal, and fast) walking speeds. Therefore, the TUG may not account for the behaviour of the biomechanical correlates of falls at slow and fast walking speeds. In contrast, the VFD employs the entire spectrum of human walking speed—from very slow to very fast—within which ADLs are likely to be executed to explain the events that occur during walking including variations in the biomechanical correlates of falls. Therefore, the VFD should provide a more holistic picture of fall discriminators, which highlights its strength.

The VFD is a simple non-invasive method of gait analysis that analyses the interaction of independent multilinear graphical regression lines of the basal gait parameters – namely, stride length, stride frequency, and stride length –in response to a neural drive [10, 11]. The gait parameters projected in the VFD form into equations very easily, which is characteristic of a neural-driven process[11].Given that gait alteration precedes falls [13, 14], it is expected that the underlying biomechanical factors should likewise find expression in the equation definition method of the VFD and justifies its useful clinical application in fall screening [26]. The strong clinical characteristics of the VFD have enabled diverse clinical studies in developed and developing countries involving plantar lipoatrophy [27], knee osteoarthritis [17], hemiparesis [28], high-heeled walking [16], and diabetes [18, 29, 30] among others [18, 29, 30]. A systematic review provided evidence that the VFD has basic scientific applications relevant to clinical studies [31], thus justifying its potential as a fall screening tool [11].

Some fall screening tools are often used in settings for which they have no basis in evidence. Thus, tools validated in acute hospitals are used in mental or community care settings/nursing homes, and vice versa. Apart from the STRATIFY score [32, 33] and the Morse Falls Scale [34, 35], most fall screening tools including the VFD, are rarely validated in more than two settings or patient cohorts. So, it is not certain what value they add to the clinical decision-making process involving screening, prevention, and management of falls. In essence, though an easy-to-use tool in fall prevention is usually recommended, but could prove misleading or detrimental to care if its reliability and validity are not evaluated in care settings of intended use. Since the TUG and VFD are commonly used in Nigeria, we sought to explore the reliability of their measurements in falls screening among community-dwelling older adults to guide practice. Therefore, the research question for this study was: What is the validity and reliability of the VFD test and TUG in fall screening compared to the gold standard (fallers), among community-dwelling older adults? The objectives of the study were to i. Determine the differences in the gait speed between fallers and non-fallers as a performance-based physical measure, ii. Determine the discrimination threshold of the VFD and TUG for fallers and non-fallers, iii. Determine the reliability and validity of the VFD and TUG in fall screening, and iv. Determine the relationship between E_1_ and gait speed in males and females.

## Methods

### Design

This is a cross-sectional observational study of 500 (220 non-fallers and 280 fallers) community-dwelling older adults aged 65-85 years. Data were collected between April 2017 – July 2018. Fallers and non-fallers were simultaneously tested with TUG and VFD, to evaluate the discriminatory power of the instruments in detecting fallers and non-fallers, and to classify future fall risks with sensitivity, specificity, and predictive values of VFD’s fall index—i.e., the value of intersection point of the stride frequency, and velocity regression lines (E_1_) measured in velots described in the VFD [10, 11]—and TUG times (measured in seconds) [9].

### Setting

This study was held at the community health centres, in Igboeze South Local Government Area (LGA), Nsukka senatorial zone, Enugu State, Nigeria comprising 10 towns, namely: Alor-Agu, Unadu, Itchi, Nkalagu-Obukpa, Ibagwa Aka, Iheakpu -Awka, Uhunowerre, Ovoko-Ulo, Ovoko-Agu, and Iheaka. These locations were selected following a rise in the number of falls involving resident older adults, who reported at the Physiotherapy unit of the University of Nigeria Teaching Hospital. On assessment, several important risk factors of falls were identified in the communities and related to poor person-environment fit/built environment (especially the lack of sidewalks and unpaved roads in the villages causing slips and trips), and poor accessibility [36, 37], dizziness/vestibular diseases, [38, 39] history of falls, gait impairments (arthritis), use of walking aids, vertigo, and use of antidepressants/sedatives [37, 40, 41], smoking/tobacco use [42], alcohol consumption [43], diabetes [40, 44], among others**. **Consequently, the research team planned a rural health education outreach programme on risk factors for falls and fall prevention strategies among older adults in the area. The health outreach programme involved the community health committees and the traditional rulers of the communities within Igboeze South LGA. Therefore, the traditional rulers convened community health fora in their respective community health centres, and the town criers were mobilized to disseminate information to all clans and villages, for at least four market days to ensure wide publicity. In addition, various age-grade groups were also invited to the health programme by the traditional rulers, through their leaders. Out of 2880 rural community dwellers who attended the health fora, eight hundred and eighty (880) were community-dwelling older adults aged ≥ 65 years and above. This group was subsequently targeted and invited to participate in this study.

### Participants

In a previous study [45], individuals classified as fallers compared to non-fallers demonstrated diminished mobility scores for all assessments. Walking coordination and walking speed over a 25-foot distance (i.e., 7.62 m which is similar to 7 m used in this study) differed between fallers and non-fallers, and the effect sizes were of moderate magnitude for TUG (d =  − 0.45) and timed walking speed (d =  − 0.46). Using the Power analysis of 80% to detect a difference between means at an effect size of 0.46, with a significance level (alpha) of 0.05 (one-tailed), a sample size of 840 was calculated using G-power software version 3.1.9.7. Considering the likelihood of attrition, 10% of the calculated sample (84) was required, however only 40 (about 5%) were available and were included in the study making a total of 880 older adults. Criteria for participation included only those who are:—(i) ≥ 65 years or older, and (ii) able to walk independently with or without assistive devices, and (iii) those who have a Mini-Mental Score Examination (MMSE) score higher than 23 [46] and (iv) not blind.

Based on self-reported history of locomotive falls, participants were categorized into 2 groups, namely: (i) fallers, and (ii) non-fallers. The fallers were operationalized in the context of this study as individuals who satisfied the fall frequency requirement of at least two falls within 12 months [47]. To identify the fallers, participants were asked the following specific questions: “*Have you fallen in the last 12 months?*”, “*If yes, how many times did you fall in the last 12 months?*”, “*Do you feel unsteady when standing or walking?*” and “*Are you worried about falling?*” The participants provided the fall frequency and confirmed by participants’ relatives/neighbours. Nevertheless, 120 participants who were unable to do so, were excluded from the study. Non-fallers were older adults who have not fallen during the past 12 months [26]. However, all those who had fallen once were categorised as a high-risk group, screened for co-morbidities of falls, and educated on fall prevention strategies. They were given some exercise regimens to strengthen their muscles and placed on the watch list and follow-up appointments. All participants provided written informed consent following approval by the Institutional Human Ethics Research Board. The study process involved three stages: obtaining informed consent, anthropometric assessment, and measurement of the risk of falls using the TUG and VFD, respectively. With the help of four trained research assistants, the 880 older adults who participated in the health forum were screened for eligibility to participate in the study. One hundred and sixty were found ineligible, thus 720 were invited to participate in the study. Out of this number, 500 (280 self-reported fallers and 220 self-identified non-fallers) individuals accepted and were requested to provide their fall history while 220 declined to participate.

The instruments used in the study included the weighing scale, stadiometer, measuring tape, and stopwatch which were used to measure the body weight, height, distance to walk/cover and walk time, respectively. The technical error measurement for weighing bathroom scale for inter-and intra-examiners for weight was 0.75% and 0.53% with inter-instrument validity showing minimal differences (range 0.1–0.4), and with inter-and intra-examiner and inter-method coefficients of variability (CV) as 0.7, 0.4 and 1.2 [48]. Bathroom scales’ measurements are precise within 0.9 kg of the actual weight of the load tested [49]. The stadiometer’s (Model: portable SECA Stadiometer 213) inter, intra-instrument and inter-instrument coefficient of reliability (R) were 99.92%, 99.93% and 99.41%, respectively. The inter-and intra-examiner and inter-instrument CVs were below 5% at 4.895%, 4.908%, and 4.943%, respectively, and therefore regarded as reliable and valid for community studies [50]. The measuring tape has high inter-observer reliability with a high intraclass correlation coefficient of 0.924 with the nearest reading of 5 mm [51]. The stopwatch (Hanbart, Germany) is impact resistant, dustproof, and water-resistant, with time intervals of 1/5 secs, displays 30 min, a resolution of 0.20 s, and has a measuring capacity of 00:30:00 h: min: sec. To eliminate inter-observer variability, only one investigator was assigned the task of evaluating the participants for the primary outcomes—TUG times and the time taken to complete a 7-m distance required for the VFD.

### An instrument for determining cognitive status

Folstein Mini-mental state examination was used to assess global cognition level, which comprised items concerning attention, language, following commands and figure copying, orientation, registration and recall for all participants. The cutoff score was < 24 [46], and none of the participants fell below the cutoff. A score of 19–23 points suggests mild dementia, whereas scores above 23 suggest normal cognition.

### Outcome measures

I. TUG test: Timed-Up-and-Go test was done with the participants sitting correctly in the chair with an armrest, with an approximate seat height of 46 cm, and arm height of 65 cm. Participants were instructed that on hearing the command “go” they were to get up and walk at a comfortable and safe pace to a line on the floor 3 m away, turn, return to the chair and sit down again. The timing started immediately after the participant got up from the chair and stopped when the participant has seated again with the back resting on the back of the chair. Each participant was required to walk through the test once before being timed to become acquainted with the procedure. The participants were required to perform the test three times, which is the number of trials needed to achieve performance stability (i.e. where no further improvement in TUG times occurs on subsequent trials) on the TUG test among older adults without hip fracture [52]. Since the TUG times improved from trial 1 to trial 3 (*P* < 0.04), and the fastest of the 3 timed trials was significantly (*P* < 0.001) faster than the other 2 trials, the fastest time of the three was used in this study as recommended by Bloch et al. [52]. Participants were also allowed to wear their regular footwear and use their customary walking aids (none, cane, walker), but no physical assistance was given. A TUG time is a time in seconds that participants needed to complete the test. Longer time indicates worse balance and mobility performance. Times under 10 s are suggestive of completely free and independent individuals; however, times ≥ 13.5 s is the cutoff point for fallers [1]. A participant’s data was collected over a 2-5 min time frame
II. VFD: The participants were required to walk a 7-m distance (measured out on the level ground using the measuring tape) barefoot or in their normal shoes. They were requested to walk the distance at five self-selected speeds: ordinary, very slow, slow, fast, and very fast, in that order. For each speed, the number of steps and time taken to complete the distance was obtained (using a Stopwatch—Hanbart Germany) and used to calculate the mean values of stride length (L), stride frequency (F), and velocity (V), for each participant as indicated in Eqs. 1–4 [10, 11]. For each participant recruited, data were collected over 2-3 min time frame.
  $$\mathrm{Strides}= \frac{No.\ of\ Steps}{2}$$
  $$\mathrm{Stride\ length }\left(\mathrm{metres}\right)= \frac{Disance}{\mathrm{No}.\ \mathrm{ of\ strides}}$$
  $$\mathrm{Stride\ frequency}= \frac{No.\ of\ Strides}{Time}$$
  $$\mathrm{Velocity}\ \left(\mathrm{V}\right)\hspace{0.17em}=\hspace{0.17em}\mathrm{Stride\ Length }\ \left(\mathrm{L}\right)\  \mathrm{x\ Stride\ frequency }\left(\mathrm{F}\right)\ \mathrm{OR\ Velocity}=\frac{Disance}{Time}$$

The regression lines of L, F, and V are known as L-line, F-line, and V-line, respectively [10, 11]. These parameters were adapted to describe the VFD [16–18, 26]. Ordinarily, the VFD consists of the graphical regression plots of the three basal gait parameters (L, F, and V) expressed in five self-selected speeds. The five self-selected speeds of walking, varying from very slow, slow, normal, fast, and very fast speeds, were serially numbered, 1-5, and assigned arbitrary units - velots. The numbers were used for the X-axis, while the numerical values of V, L, and F, were used on the Y-axis. These lines make up the primary features of the VFD [10, 11]. The point of equality for the numerical values of velocity and stride frequency (E_1_) marked the upper limit of very slow speed and a speed transition to the path of minimal energy trajectory [16–18, 26]. Similarly, the point of equality for the numerical values of velocity and stride length (E_2_) marked the upper limit of normal speed and a speed transition to fast walking speed. Eke-Okoro [26] demonstrated that 3.5 velots is the value of E_1_ on the VFD of fallers, which discriminated them from non-fallers (≤3.5 velots) and therefore has a diagnostic application. Consequently, Eke-Okoro [26] predicted that the critical point for the onset of fall is E_1_≥3.5 velots.

### Covariates

Information on age and gender were obtained by self-report. Co-morbidities were ascertained by self-report questionnaires. Comorbidities were defined as a history of the potential confounding effects of diseases of orthopaedic or neurological nature including chronic co-morbidities (history of dizziness, vestibular disease, and arthritis). The other covariates were the cardiovascular risk factors including diabetes, obesity, smoking, alcohol consumption, gender, and the use of anti-anxiety drugs [53–59], especially consumption of alcohol within 72 h of gait recording [56, 60, 61], and use of sedatives and anti-anxiety drugs within 72 h of gait recording [56, 60]. Some of the identified anti-anxiety drugs are presented below using their codes according to the Anatomical Therapeutic Chemical (ATC) classification system [62]. They included drugs acting on the nervous system (Group N) including N05A – Anti-psychotics, N05BA – Anxiolytics (benzodiazepine-derivatives), N05CD-Hypnotics, and sedatives (benzodiazepine-derivatives). Alcohol consumption was dichotomized and defined as weekly alcohol consumption of ≥ 11 units for men and ≥ 8 units for women. Body mass index (BMI), was determined as weight in kilograms divided by the square of height in meters, and categorized according to the National Institutes of Health obesity standards < 18.5 = underweight, 18.5–24.9 = normal weight, 25.0–29.9 = overweight, and > 30 = obese [63].

### Data analysis

Data collected for this study were presented in tables. Participants’ characteristics, as well as means and SD values for continuous variables and percentages for categorical variables, were determined for males and females. Data were principally analyzed for males and females combined, but analyses were repeated for males and females separately. Differences in gait parameters between fallers and non-fallers were determined with and without adjustment for the potential confounding effects of the above covariates.The normality of distribution of the basal gait parameters (stride velocity, stride frequency, and stride length), was confirmed by the Kolmogorov–Smirnov test. Descriptive statistics and an independent t-test were used to analyze the biodata, anthropometric indices, mean differences in VFD performance (absolute and adjusted), and physical characteristics between fallers and non-fallers. McNemar test was used to determine the classification accuracy of the VFD relative to the TUG, while weighted Kappa was used to determine the level of agreement between the TUG and VFD in screening out fallers from non-fallers. For all kappa statistics, κ values were interpreted as follows: below 0 as less than chance, 0.01–0.20 as slight, 0.21–0.40 as fair, 0.41–0.60 as moderate, 0.61–0.80 as good, and above 0.80 as very good levels of agreement [64]. The sensitivity and specificity of the VFD and TUG were also determined.The estimated population midpoints and 95% confidence intervals were calculated for: (i) prevalence of the condition; (ii) test sensitivity (conditional probability that the test will be positive if the condition is present); (iii) test specificity (conditional probability that the test will be negative if the condition is absent); (iv) predictive values of the test (probabilities for true positive, true negative, false positive, and false negative); and, (v) positive and negative likelihood ratios.

After stratification based on sex, weighted linear regression analysis was applied to explore the relationship between E_1_ and gait speed as a performance-based physical measure. The distributions of E_1_ in both men and women were right-skewed. Consequently, it was considered appropriate to use natural-log-transformed values, which gave the best-fitting model for analysis in which the E_1_ values were treated as continuous variables. For both males and females, standard-deviation scores of E_1_ were obtained from the formula (Xi-Xm) ÷ SD, where Xi was the natural-log-transformed E_1_ in the individual male/female subject, Xm is the mean natural-log-transformed E_1_ in the male/female subjects, and SD the standard deviation of the natural-log-transformed E_1_ in the male/female subjects. With this calculation, it was possible to determine the change in the gait speed for each increment of 1SD in the natural-log-transformed E_1_. The relations of E_1_ to gait speed were also estimated with a quartile-based analysis by dividing E_1_ values into quartiles with subjects in the lowest quartile as the reference group.Comorbidities were assessed by referring to the self‐reported physician's diagnosis and included dizziness, vestibular disease, diabetes, alcohol consumption, and arthritis. An extended-model approach was applied for covariates adjustments: Model 1 = Age, weight, smoking status, alcohol consumption, and use of walking devices; Model 2 = Model 1 + co-morbidities (dizziness, vestibular diseases, diabetes, and arthritis); Model 3 = Model 2 + markers of cardiovascular risk (natural-log-transformed levels of the use of anti-anxiety drugs, gender). Body mass index categories (BMI) were also controlled in the association between E_1_ and gait velocity (Model 4) to observe the possible change of association.

Statistically significant associations were identified at a 5% significance level, but a Bonferroni correction was also applied to enable the identification of significant associations after allowance for multiple comparisons. Receiver operating characteristic (ROC) curves were calculated to analyze the discriminant validity of the VFD and TUG. ROC computes the true positive and false positive for each test value and plots them on a curve. The area under the ROC curve (AUC) is interpreted as a measure of the classification quality of the test. The AUC values range from 0 and 1, with higher values indicating better classification accuracy. As much as the AUC value is closer to 0.5, the poorer the classification accuracy of the test is because the value of 0.5 corresponds to a random classification. The overall prediction rate (i.e. total number of true positives + true negatives/total sample) of the VGD and TUG were also determined as a measure of their classification accuracy. XLSTAT-BIOMED statistical software was used for this analysis, which computes the *p*-value with a logistic regression model.A p-value < 0.05 implies that the logistic regression classifies the fallers based on the empirical data better than by chance. The 95% confidence interval (CI) estimates the interval of the population parameter out of the study data. For any diagnostic or screening tool, the lower bound of the 95% CI of the ROC curve, should be greater than 0.5 if not, the risk that the real population estimate is not better than a random classification is too high.

## Results

**Fig. 1:**
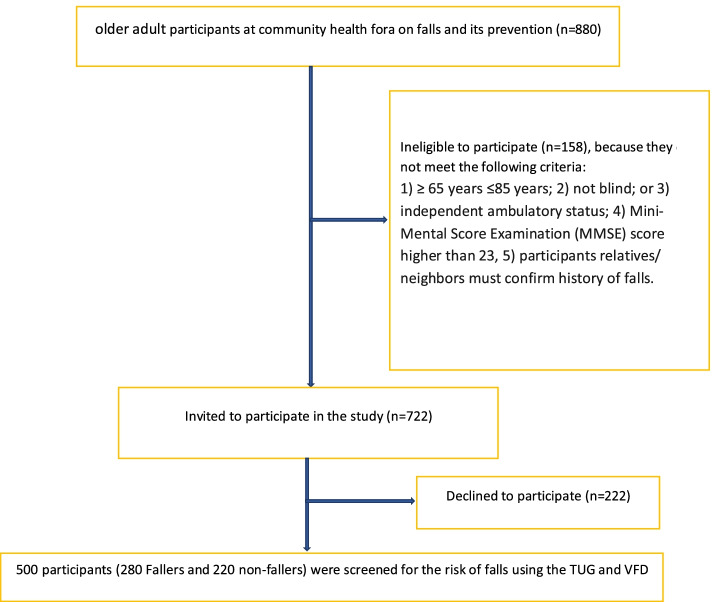
Design and flow of participants through the study

The participant’s flow through the study is presented in Fig. 1.

### Characteristics of participants

The descriptive statistics for the biodata and anthropometric indices for the community-dwelling older adult fallers, (*n* = 280; comprising 150 males and 130 females) and non-fallers (*n* = 220; comprising 100 males and 120 females) are presented in Table 1. Relatively, the non-fallers were older, taller, and heavier than the fallers, but these differences were not significant (*p* > 0.05). However, the fallers have significantly higher BMI values than the non-fallers (*p* = 0.0001). The sex distribution of the characteristics of the participants is presented in Table 2. The mean age and age range for males and females were 69.8 years (age range: 65 to 85 years), and 74.48 years (age range; 65 to 85 years), respectively. A greater number of the males were fallers but the proportional difference was not significant (Z = 1.80; *p* = 0.72) (Table 1). Compared to the female fallers, the male fallers had significantly (*p* < 0.05) higher fasting plasma glucose (*p* =  < 0.001), proportion of current smokers/tobacco users (Z = 7.67; *p* =  < 0.00001), alcohol intake (Z = 7.97; *p* =  < 0.00001), anti-anxiety medications use (Z = 6.28; *p* =  < 0.00001), cases of diabetes (Z = 3.49; *p* = 0.00048), vestibular diseases (Z = 3.87; *p* = 0.0001) with higher but insignificant cases of dizziness (Z = 1.92; *p* = 0.055). The female fallers had significantly higher cases of arthritis (Z = -1.99; *p* = 0.047). (Table 2).

**Table 1:** Biodata, and anthropometric indices for fallers (*n* = 280) and non-fallers (*n* = 220)

Variables	Participants (*n* = 500)	Non-fallers (***n*** = 220)	Fallers (***n*** = 280)	Mean difference	*p*-value
**Continuous variables X(SD)**					
Age (years)		72.14(4.54)	70.04(3.76)	2.10	0.35
Height (cm)		164.00(6.78)	160.43(8.13)	3.57	0.10
Weight (Kg)		73.00(11.29)	70.61(17.23)	2.39	0.57
BMI (Kg/m^2^)		25.86(0.23)	27.58(0.26)	1.72	0.0001**
**Categorical variables N(%)**					
Sex ●Males	250 (50.0)	100 (45.45)	1503.57)		
●Females	250 (50.0)	120 (54.54)	130 (46.43)		.07
●Total	500 (100.0)	220 (100)	280 (100)		

Values were expressed as mean (X), standard deviation (SD), number (N) and percentage (%), ** indicates p ≤ 0.0001

**Table 2:** Gender distribution of the risk factors for falls among the participants (*N* = 500)

Characteristics	Males	Females	Mean difference	Z	*P*-value
**Continuous variables X(SD)**					
Fasting blood sugar(mg/dL)					
●Fallers & Non-fallers	102.0 (0.5)	98.4 (0.5)	3.6		< 0.001**
**Categorical variables,N (weighted %)**					
Current smokers/tobacco users^a^					
●Fallers	110	30		7.67	< .00001****
●Non-fallers	30	0			
**Total n(/%)**	140 (50.0)	30(10.71)			
Moderate or higher weekly alcohol intake^b, c^					
●Fallers	120	40		7.97	< .00001****
●Non-fallers	20	10			
** Total n(/%)**	140 (50.0)	50 (17.86)			
Number on anti-anxiety Drugs Medication^c^					
●Fallers	90	30		6.28	< .00001****
●Non-fallers	40	20			
** Total n(/%)**	130 (46.43)	50 (17.86)			
Diabetes^a^					
●Fallers	60	30		3.49	.00048***
Non-fallers	30	0			
** Total n(/%)**	90 (32.14	30 (10.71			
Dizziness^a^					
●Fallers	90	70		1.92	.055
Non-fallers	20	10			
** Total n(/%)**	110 (39.29)	80 (28.57)			
Vestibular disease^a^					
●Fallers	50	20		3.87	.0001***
Non-fallers	0	0			
** Total n(/%)**	50 (17.86	20 (7.14)			
Arthritis^a^					
●Fallers	60	80		-1.99	.047*
Non-fallers	20	40			
** Total n(/%)**	80 (28.57)	120 (42.86)			

Values were expressed as mean (SD) and number (weighted %)

^a^Number and percentage

^b^Defined as weekly alcohol consumption of ≥ 11 units for men and ≥ 8 units for women

^c^Variable was positively skewed; median and inter-quartile range presente

^**^ indicates p ≤ 0.001

^***^ indicates p ≤ 0.0001

### Differences in gait speed between fallers and non-fallers

The non-fallers mean velocity (V = 0.44 ± 0.14 m/s) was significantly higher (*p* = 0.05) compared to fallers (V = 0.38 ± 0.06 m/s) at the slow walking speed (Table 3) otherwise both groups were similar across the other walking speeds.

**Table 3:** Basal gait parameters and phases of stride for fallers (*n* = 280) and non-fallers (*n* = 220)

Variables	Velotype 1	Velotype 2	Velotype 3	Velotype 4	Velotype 5
	Mean SD	Mean SD	Mean SD	Mean SD	Mean SD
Stride length (m)					
●Fallers	0.56 0.15	0.63 0.16	0.71 0.15	0.83 0.19	0.96 0.24
●Non-fallers	0.57 0.17	0.64 0.18	0.72 0.22	0.80 0.27	0.97 0.29
* p*-value	0.86	0.79	0.86	0.64	0.92
Stride frequency					
(strides/s)					
●Fallers	0.58 0.06	0.62 0.10	0.63 0.16	0.80 0.14	0.86 0.16
●Non-fallers	0.55 0.07	0.61 0.12	0.64 0.18	0.80 0.17	0.85 0.17
* p*-value	0.10	0.89	0.39	1.00	0.84
Velocity (m/s)					
●Fallers	0.31 0.07	0.38 0.06	0.49 0.08	0.66 0.09	0.88 0.34
●Non-fallers	0.31 0.09	0.44 0.14	0.50 0.12	0.65 0.10	0.88 0.39
* p*-value	0.92	0.05^a^	0.74	0.73	1.00

^a^ indicates *p* < 0.05; Velotype 1 – very slow walking speed; Velotype 2 = slow walking speed; Velotype 3 = normal walking speed; Velotype 4 = fast walking speed; Velotype 5 = very fast walking speed

### Discrimination threshold of the VFD and TUG

**Fig. 2:**
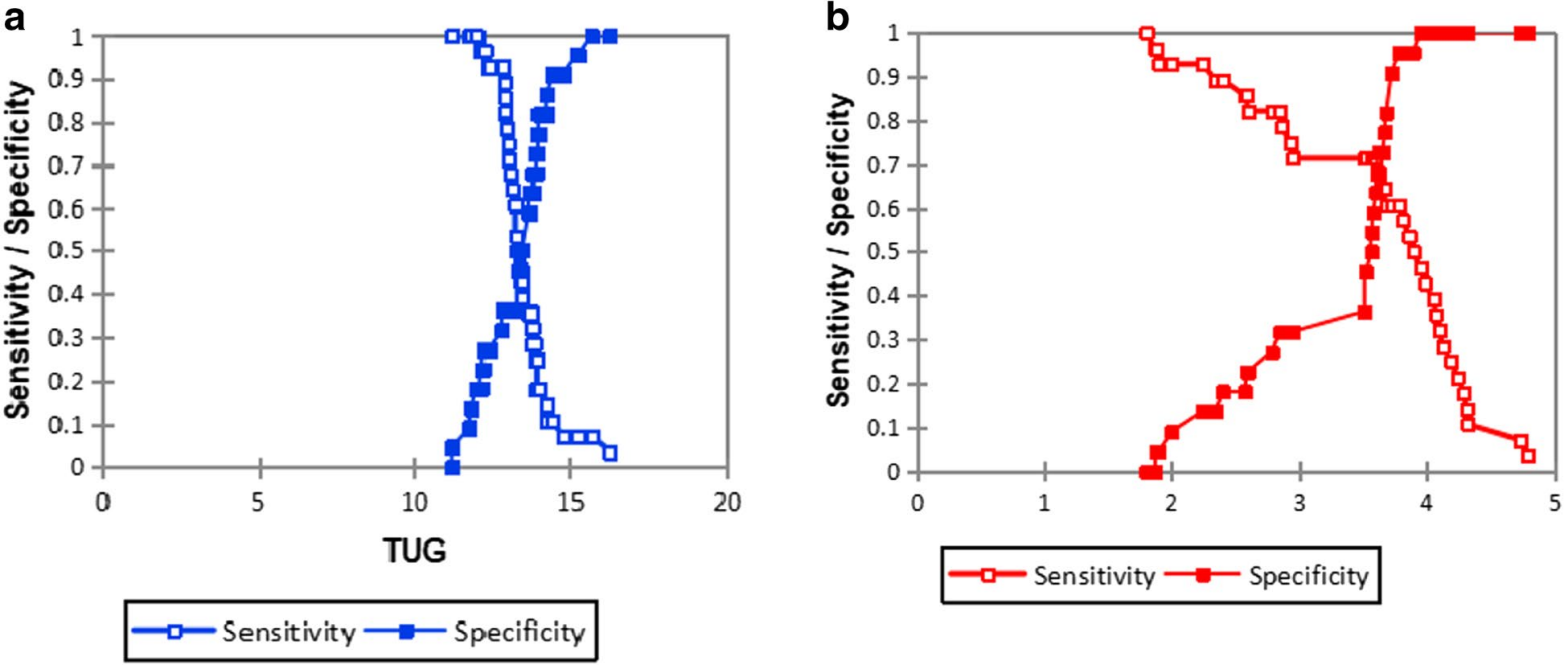
**a** The optimal categorizations for discrimination between fallers and non-fallers in relation to sensivity + specify/ TUG times. TUG = Timed-up-and-go-test. **b** The optimal categorisations for discrimination between fallers and non-fallers in relation to sensitivity and specificity/VFD E_1_. VFD = Velocity field diagram

The optimal categorizations for discrimination between fallers and non-fallers using the VFD (Fig. 2a) were ≥ 3.78 versus ≤ 3.78 for E_1_ (fallers and non-fallers prevalence 60.71% versus 95.45%, respectively), with a classification accuracy of 0.76.For the TUG, the area under the curve was 0.53, with a 95% CI = [0.35, 0.70], which is not significant (*p* = 0.76). The optimal categorizations for discrimination between fallers and non-fallers (Fig. 2b), were ≥ 12.81 s versus ≤ 12.81 for TUG times (fallers and non-fallers prevalence 92.86% versus 36.36%, respectively), and with a classification accuracy of 0.68.

### Reliability and validity of the VFD and TUG in fallers and non-fallers discrimination

**Fig.3:**
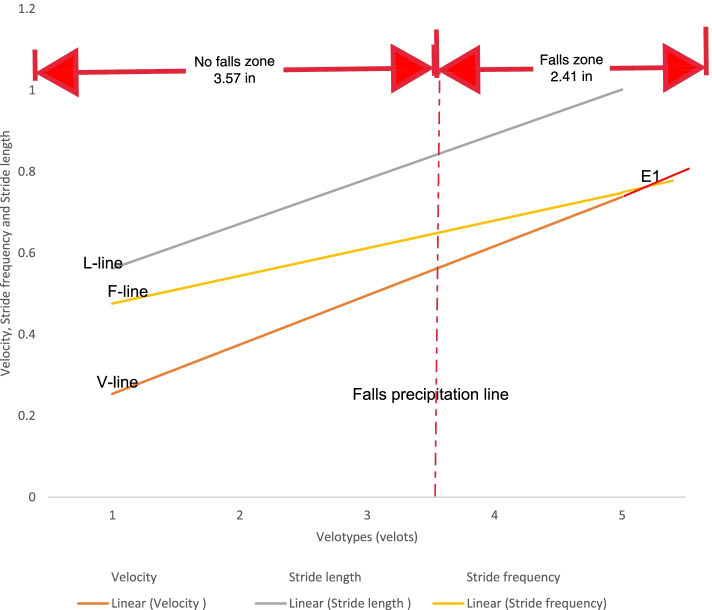
Velocity field diagram of a faller. 1 = Very slow walking; 2 = slow walking; 3 = normal walking; 4 = fast walking; 5 = very fast walking L-Line = regression line of stride lenth; F-Line = regression line of Stride frequency; V-Line = regression line of velocity; E_1_ of fallers is 3.5 velots = fall precipitation line

**Fig. 4:**
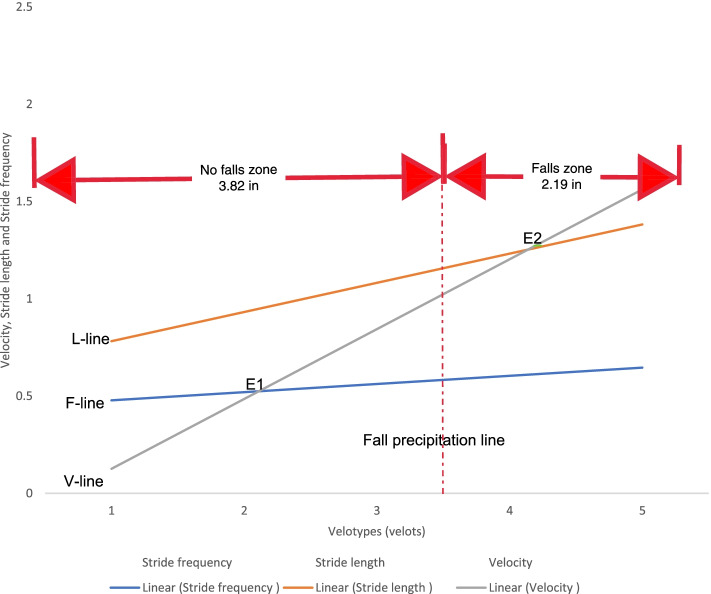
Velocity field Diagram of a non-faller. 1 = Very slow walking; 2 = slow walking; 3 = normal walking; 4 = fast walking; 5 = very fast walking  L-Line = regression line of stride length; F-Line = regression line of Stride frequency; V-Line = regression line of velocity; E_1 _of fallers is 3.5 velots = fall precipitation line

**Fig. 5:**
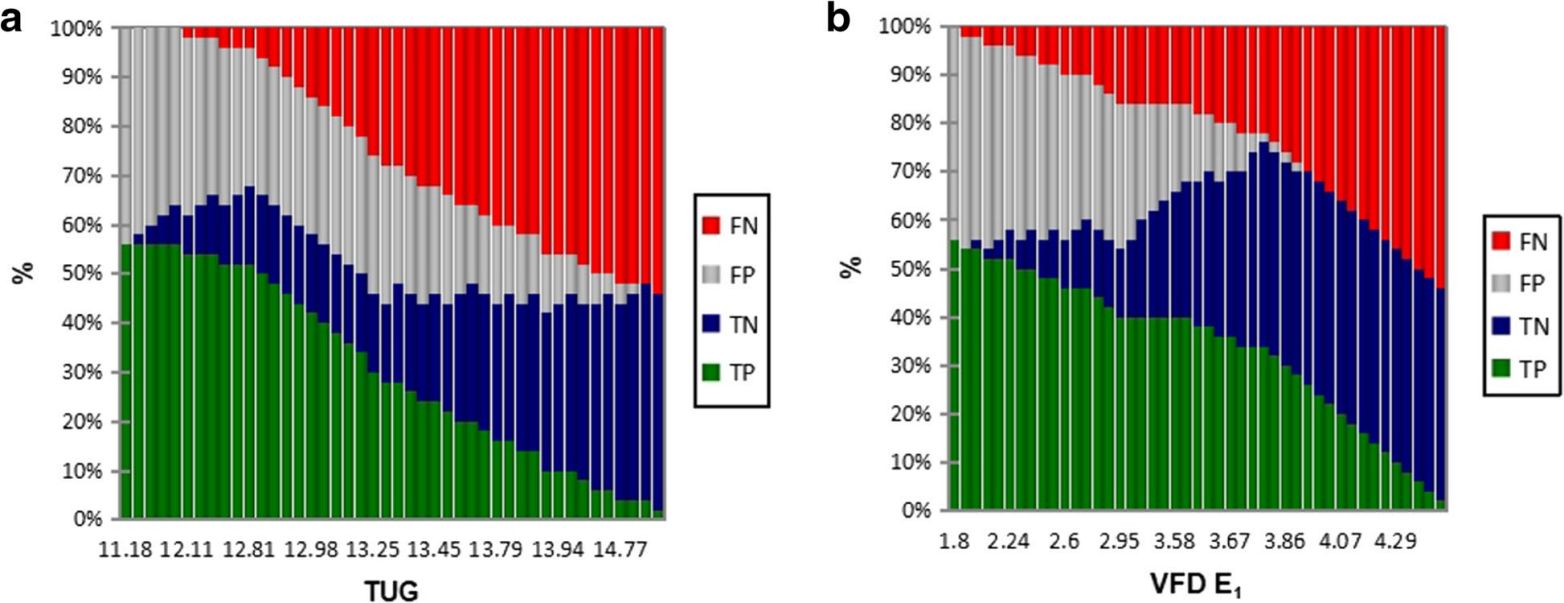
**a** The evolution of counting-True positive (TP), True negative (TN), False positive (FP), False negative (FN) in relation to thresholds of TUG. TUG = Timed-up-and-go-test. **b**. The evolution of counting-True positive (TP), True negative (TN), False positive (FP), False negative (FN) in relation to thresholds of VFD E_1_. VFD = Velocity field diagram

**Fig. 6:**
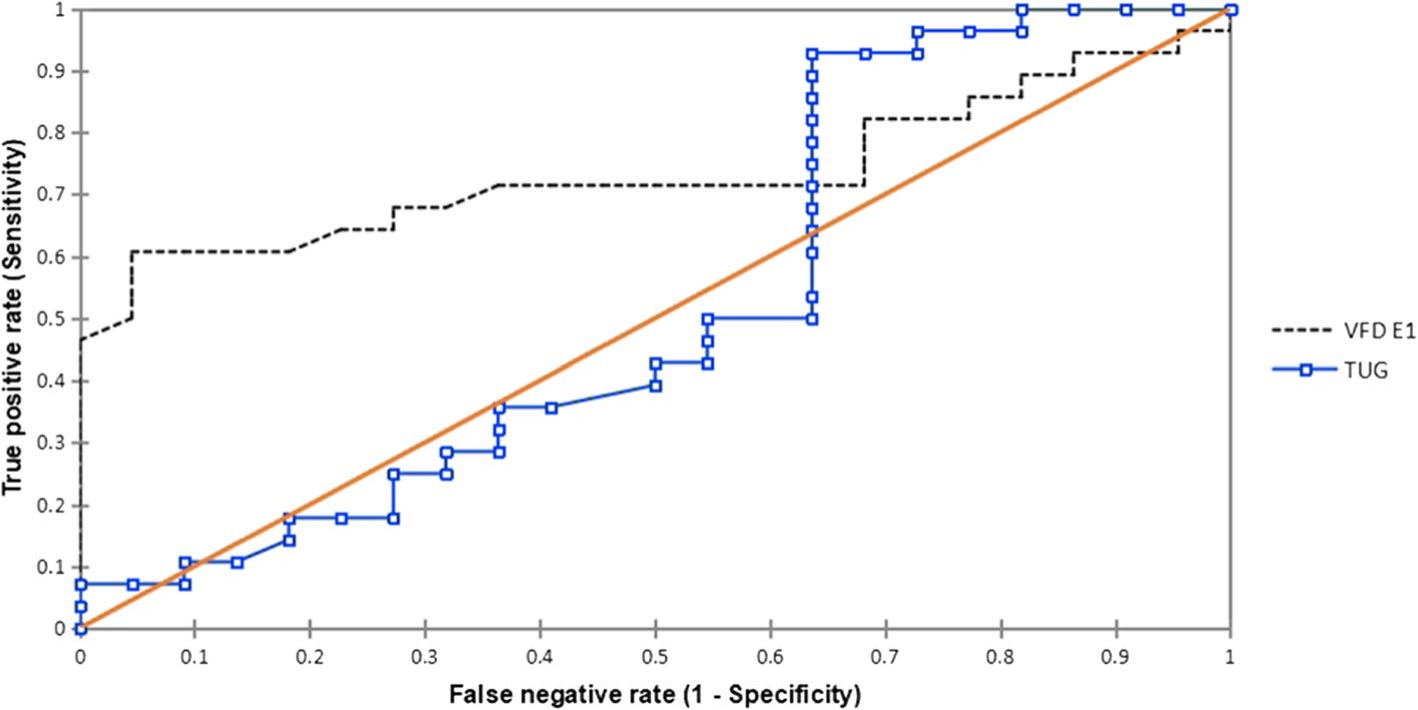
Compasiron of the AUC of the ROC curves for TUG and VFD. TUG = Timed-up-and-go-test; VFD = Velocity field diagram, AUC = Area under the curve; ROC = Receiver operating characteristics

VFD (Figs. 3–4) and TUG were separately used to discriminate fallers from non-fallers in a group of community-dwelling older adults. When applied to the same population of 280 older adult fallers (Figs. 5a, and b), the TUG returned 110 (39.29%) positive test results, whereas the VFD returned 200 (71.43%) positive test results (Table 4). A similar assessment among 220 non-fallers showed that the TUG returned 130 (59.09%) negative test results whereas the VFD returned 60 (27.27%) negative test results (Table 4). Cohen’s quadratic weighted Kappa, (K = 0.2804 SE 0.12; 95%:CI: 0.05- 0.51) suggests a minimal level of agreement between both instruments.It was shown (Table 4) that the sensitivity, specificity, positive predictive value, and negative predictive value of the VFD were 71%, 27%, 55%, and 42%, respectively. This compares favorably with 39%, 59%, 55%, and 43% recorded for the TUG in the same population. Thus, the VFD maintained a higher overall prediction rate (i.e. total number of true positives + true negatives/total sample) 260/500 (52%) compared to the TUG 240/500 (48%). ROC curves (Fig. 6) were used to explore the ability of the VFD and TUG to discriminate between fallers and non-fallers. The ROC area under the curve was 0.74 (95%CI 0.597, 0.882) for VFD (Table 5); which was significant (*p* = 0.001) and is suggestive of fair discrimination between fallers and non-fallers. The ROC analysis for the entire sample (male and female combined), (Table 6**)** also showed the sensitivity and the specificity of the TUG and the VFD for different thresholds. With regards to the desired balance between sensitivity and specificity, an appropriate cutoff point could be selected. A higher cutoff point increases the specificity, and a lower cutoff point increases the sensitivity.

**Table 4:** Sensitivity and specificity of the Velocity field diagram and Timed-up-and-go-test in fall prediction among fallers (*n* = 280) and non-fallers (*n* = 220)

				95%CI		
Variable	Non-Fallers n(%)	Fallers (gold standard) F(%)	Estimated values	Lower limit	Upper limit	Diff CI
No. of participants that tested positive						
●VFD	160(72.7)	200(71.4)				
●TUG	90(40.9)	110(39.3)				
No. of participants that tested negative						
●VFD	60(27.3)	80(28.6)				
●TUG	130(59.1)	170(60.7)				
Prevalence			0.56	0.41	0.70	0.29
Sensitivity						
●VFD			0.71	0.52	0.85	0.37
●TUG			0.39	0.22	0.59	0.37
Specificity						
●VFD			**0.27**	0.20	0.57	0.37
●TUG			0.59	0.37	0.79	0.42
PPV						
●VFD			**0.55**	0.56	0.83	0.26
●TUG)			**0.55**	0.27	0.55	0.28
NPV						
●VFD			**0.42**	0.19	0.71	0.52
●TUG			**0.43**	0.26	0.62	0.36
**likelihood Ratios:**						
Positive[C]						
●VFD			1.12	0.69	1.39	0.70
●TUG			0.79	0.49	1.90	1.41
Negative[C]						
●VFD			0.79	0.46	2.30	0.84
●TUG			1.21	0.72	1.47	0.75

**Table 5:** Comparison of the Area Under the ROC Curve for Velocity field diagram and ‘Timed-Up-and-go test

tool	**Sensitivity (% Fallers)**	**Specificity (%Nonfallers)**	**Overall Prediction**	**Area Under the ROC Curve**			**Comparison of the AUC to 0.5**			
				AUC	SE	95% CI	Difference	z (Observed Value)	p	95% CI between AUC and 0.5 (Two-tailed test)
TUG	110/280 (39.28%)	130/220 (59.09%)	240/500 (48%)	0.53	0.09	[0.35, 0.70]	0.03	0.30	0.76	[0.147,0.20]
VFD	200/280 (71.42%)	60/220 (27.27%)	260/500 (52%)	0.74	0.07	[0.60, 0.88]	0.24	3.30	0.001*	[0.091,0.38]

^*^ indicates p ≤ 0.001; AUC = Area under the curve; SE = Standard error; 95% CI = 95% confidence interval; z (Critical value) = 1.96

**Table 6:** Sensitivity and Specificity of the TUG and VFD for different thresholds

Screen tool	Sensitivity	Specificity	PPV	NPV	Accuracy
TUG **cutoff time (s)**					
≤ 11.77	1.00[0.83, 1.00]	0.09[0.02, 0.29]	0.52	1.00	0.60
** ≤ 12.88**	**0.86[0.68, 0.95]**	**0.36[0.20, 0.57]**	**0.57**	**0.72**	**0.64**
≤ 13.25	0.54[0.36, 0.70]	0.36[0.20, 0.57]	0.46	0.44	0.46
≤ 13.72	0.36[0.21, 0.54]	0.64[0.43, 0.80]	0.50	0.50	0.48
≤ 13.98	0.18[0.08, 0.36]	0.82[0.61, 0.93]	0.50	0.50	0.46
VFD **cutoff values for E**_**1**_					
≤ 1.80	1.00[0.88, 1.00]	0.00[0.00, 0.18]	0.50	0.00	0.56
≤ 2.57	0.86[9.68, 0.95]	0.23[0.01, 0.44]	0.51	0.57	0.56
≤ 2.93	0.75[0.56, 0.88]	0.32[0.16, 0.53]	0.52	0.56	0.56
** ≤ 3.68**	**0.61[0.42, 0.76]**	**0.82[0.61, 0.93]**	**0.77**	**0.68**	**0.70**
≤ 4.05	0.39[0.24, 0.58]	1.00[0.82, 1.00]	1.00	0.62	0.66
≤ 4.29	0.18[0.08, 0.36]	1.00[0.82, 1.00]	1.00	0.55	0.54

*Note*. The values in brackets represent 95% confidence intervals. *TUG* Timed Up and Go Test, *VFD* Velocity field diagram

Relative to the TUG, the VFD returned a positive test result for 70 of the 110 positive test results from TUG, so the estimated sensitivity of the VFD relative to the TUG is 70/110 × 100% = 63.63%. In contrast, the VFD returned negative test results for 40 out of 170 negative test results from TUG, so the estimated specificity of VFD relative to the TUG is 40/170 × 100% = 23.53%. McNemar test showed (Table 7) a significant (*p* < 0.05) concordance, (McNemar test: Pa = 20/28 = 0.7143, Pb = 11/28 = 0.39, Pa/Pb = 0.32, *p* = 0490; odds ratio larger/smaller) = 3.25; 95% CI: 0.97—1.06), and a small confidence interval when the classification accuracy of the VFD was compared to the TUG.

**Table 7:** Discriminatory ability of the velocity field diagram relative to the reference standard (TUG) tested among fallers (*n* = 280)

TUG						
VFD	Test condition	Positive	Negative	Total	Estimated sensitivity of VFD	estimated specificity of VFD
	Positive	70	130	200	63.63%	23.53%
	negative	40	40	80		
	Total	110	170	280		

*VFD* Velocity diagram; VFD correctly identified 70 of the 110 positive samples, so the estimated sensitivity of VFD is 70/110 × 100% = 63.63%. Four of the 170 negative samples were correctly identified, so the estimated specificity of VFD is 40/170 × 100% = 23.53%.; McNemar test: Pa = 200/280 = 0.7143, Pb = 110/280 = 0.3929, Pa/Pb = 0.3214, p = 0490; odds ratio larger/smaller) = 3.25; 95% CI: 0.97—1.06

### Sex-related models of the relationship between E_1_ and gait speed

E_1_ was higher in males than females. The association between E_1_ and gait velocity is presented in Table 8. The cut-off values for E_1_ quartiles among the males were: quartile 1 (< 1.80), quartile 2 (1.80–2.51), quartile 3 (2.52–3.69), and quartile 4 (> 3.69); while among the women they were quartile 1 (< 1.48), quartile 2 (1.48–2.28), quartile 3 (2.29–3.52), and quartile 4 (> 3.52). The males were similar to women in terms of age, weight, and gait velocity. E_1_ values were inversely associated with velocity among the men. After adjustment for age, weight, alcohol consumption, and use of walking devices, each increment of 1SD in the E_1_ values was associated with a 0.049 m/sec decrease (*p* = 0.011) in velocity (Table 8).

**Table 8:** Association between E_1_ and gait speed in male and female

	**Models with E**_**1**_** as a continuous variable**							
	**Men**	β* (SE)	***P* value**		**Women**	β* (SE)	***P* value**	
Model 1		-0.038 (0.015)	0.017*			-0.019 (0.017)	0.172	
Model 2		-0.038 (0.017)	0.015*			-0.016 (0.016)	0.278	
Model 3		-0.046 (0.018)	0.013*			-0.014 (0.018)	0.372	
Model 4		-0.049 (0.017)	0.011*			-0.019 (0.014)	0.369	
	**Models with E**_**1**_** by increasing quartiles**							
	**Men**				**Women**			
	**Quartile comparison**	**β†** **(SE)**	***P* value**	**P for trend**	**Quartile comparison**	**β†** **(SE)**	***P* value**	**P for trend**
Model 1	Q2 v.s. Q1	-0.017 (0.037)	0.638	0.038*	Q2 v.s. Q1	0.049 (0.027)	0.092	0.464
	Q3 v.s. Q1	-0.064 (0.048)	0.146		Q3 v.s. Q1	-0.032 (0.031)	0.335	
	Q4 v.s. Q1	-0.078 (0.033)	0.078		Q4 v.s. Q1	-0.001 (0.035)	0.978	
Model 2	Q2 v.s. Q1	-0.011 (0.055)	0.790	0.056	Q2 v.s. Q1	0.052 (0.027)	0.075	0.531
	Q3 v.s. Q1	-0.059 (0.039)	0.197		Q3 v.s. Q1	-0.025 (0.030)	0.403	
	Q4 v.s. Q1	-0.058 (0.034)	0.078		Q4 v.s. Q1	0.006 (0.038)	0.908	
Model 3	Q2 v.s. Q1	-0.015 (0.029)	0.567	0.024*	Q2 v.s. Q1	0.058 (0.029)	0.048*	0.705
	Q3 v.s. Q1	-0.046 (0.031)	0.120		Q3 v.s. Q1	-0.022 (0.033)	0.508	
	Q4 v.s. Q1	-0.072 (0.035)	0.030*		Q4 v.s. Q1	0.016 (0.037)	0.707	
Model 4	Q2 v.s. Q1	-0.008 (0.036)	0.786	0.019*	Q2 v.s. Q1	0.052 (0.029)	0.059	0.673
	Q3 v.s. Q1	-0.048 (0.038)	0.167		Q3 v.s. Q1	-0.022 (0.031)	0.565	
	Q4 v.s. Q1	-0.063 (0.023)	0.024*		Q4 v.s. Q1	0.009 (0.036)	0.809	
**E**_**1**_	**Men**				**Women**	**Mean Difference**	***P*-value**	
Overall Mean	3.75 ± 1.37				3.50 ± 1.25	0.25	0.044*	

^*^ Indicates *p* ≤ 0.05,

Adjusted covariates: Model 1 = Age, weight, smoking status, alcohol consumption, and use of walking devices; Model 2 = Model 1 + co-morbidities (dizziness, vestibular diseases, diabetes, and arthritis)

Model 3 = Model 2 + markers of cardiovascular risk (natural-log-transformed levels of the use of anti-anxiety drugs, gender)

Model 4 = Model 3 + BMI categories

^*^ Parameter estimates (β) can be interpreted as differences in mean gait speed (m/sec) for each increment

of one standard deviation in the log-transformed E_1_ among men (or women)

^†^ Parameter estimates (β) can be interpreted as differences in mean gait speed (m/sec) compared male (or female)

subjects in the 2nd, 3rd, and 4th quartiles of E_1_ to those in the lowest quartile

Abbreviations: E_1_ – Equality point for the regression lines of velocity and stride frequency on the VFD; SE, standard error

The cut-off values E_1_ quartiles among the men were: quartile 1 (< 1.80), quartile 2 (1.80–2.51), quartile 3 (2.52–3.69),

and quartile 4 (> 3.69); while among the women the cut-off values were: quartile 1 (< 1.48), quartile 2 (1.48–2.28),

quartile 3 (2.29–3.52), and quartile 4 (> 3.52)

Additional adjustment of covariates including chronic co-morbidities and markers of cardiovascular risk (diabetes, smoking, alcohol consumption, gender, and the use of anti-anxiety drugs) did not alter the association among men (Model 2 and Model 3). In the fully-adjusted model where BMI was additionally adjusted (Model 4), the negative association between E_1_ and velocity among men was unchanged (β coefficient -0.049, *p* = 0.011). However, there was no association between E_1_ and velocity in the females, and a subsequent division of E_1_ into quartiles showed that velocity among the females in the highest E_1_ quartile was 0.068 m/sec less than that for males in the lowest quartile after adjustment for Model 1 covariates (significant trend across E_1_ quartiles with *p* = 0.038). Likewise, supplementary adjustment for additional covariates (Models 2 to Model 4) did not alter the inverse association between E_1_ and velocity in the males, in the quartile-based analyses. Overall, no clear trend was established between E_1_ quartiles and velocity in the females. Stratified by gender, adjusted means of velocity based on different E_1_ quartiles were obtained from the full-adjusted regression models.

## Discussion

The main objective of this study was to examine whether the VFD is a valid instrument for determining the fall status of community-dwelling elderly people and to compare its discriminatory ability to the TUG as a more established fall screening tool. The ROC curves were used to explore the ability of the VFD and TUG to discriminate between fallers (gold standard) and non-fallers, respectively. The VFD’s ROC has a high AUC (0.74, *p* = 0001; 95%CI: 0.597, 0.882) which suggests that it has a fair discriminatory power, and could serve as a more accurate fall screening tool than the TUG (AUC = 0.53: 95% CI:0.353, 0.700, *p* = 0.762).

An ideal diagnostic test has high sensitivity and specificity for detecting the condition of interest [65]. The VFD’s higher sensitivity (ability to rule out) implies that it returned fewer false-negative results than the TUG, while its narrower 95% CI shows a greater precision in its measurement than the TUG. Notwithstanding that the PPV of both tools were the same (55%), the VFD’s 71% sensitivity implied that nearly three out of four older adult fallers would test positive unlike one out of four older adult fallers recorded for the TUG (39% sensitivity). However, the reverse is the case for specificity (ability to rule in) as the TUG showed a slightly greater ability in identifying non-fallers than the VFD. The TUG's NPV (43%) was thus slightly greater than the VFD (42%). Given that the PPV and NPV are affected by disease prevalence [66], the likelihood ratios (LR) were therefore determined since it provides a statistic about the extent to which the test reliability is independent of disease/outcome prevalence [67]. The VFD’s greater weighted LR + and LR- compared to the TUG implies that its reliability in determining the older adults’ fall status is more independent of disease/outcome prevalence than the TUG. The differences in the sensitivity and specificity of the VFD and TUG could explain why the Cohen’s quadratic weighted Kappa, (K = 0.2804 SE 0.12; 95%:CI: 0.05- 0.51) showed a minimal agreement between both tools in fall discrimination as only 4–15% of the data are reliable if the TUG were to be a reference standard.

The fallers’ and non-fallers’walking speeds did not differ except at slow walking suggesting that the basal gait parameters of fallers were unable to compensate for one another at slow walking speeds, as explained in the mathematical relationship: velocity = stride length x stride frequency. Ordinary/normal walking speed marks the path of minimal energy trajectory (which signifies the efficient stability region) whereby the cyclic lower limb swing frequency attains its natural pendulum rate [17, 68]. Therefore, at slower speeds below the normal walking speed, muscle contraction and greater energy expenditure would be required to deliberately retard the limb swing below its natural pendulum rate [68], and therefore signifies the inefficient stability region. A locomotor system with weakened muscles would be challenged at slow walking speed and therefore unable to increase the stride length to compensate for a reduction in stride frequency. A shorter stride length implies a reduction in pelvic rotation and difficulty in lowering the vertical projection of the body’s centre of gravity required to optimize stability at the double-support phase of the stride [18, 69]. The above gait features of the older adult fallers imply a tendency towards instability that could be aggravated by disequilibrium with repetitive stepping cycle [70] and predisposing to locomotive fall. Therefore, the point of intersection of the regression lines of stride frequency and gait velocity, defined as E_1_ on the VFD, would likely vary in fallers and non-fallers. This is the basis on which E_1_ is considered a fair discriminator of fallers from non-fallers. Unlike the VFD, the TUG screens for fallers on the basis of their ambulatory performance at only the normal but not fast and slow walking speeds. Therefore, the TUG would be deficient in characterizing the biomechanical correlates of falls in the activities of daily living throughout the entire spectrum of human walking speeds within which locomotive falls may occur.

The normal walking speeds of older adult fallers (0.49 m/s) and non-fallers (0.50 m/s) were below 1 m per second and were categorised as slow walking speeds [71]. Slow speed in this population could arise from antalgic gait due to arthritis [17] that was prevalent in 140 (28%) fallers and 60 (12%) non-fallers. Additionally, racial factors contribute to slow speed which was prevalent in 94% of older African-Americans compared to 85% of older non-Hispanic Whites (*p* < 0.001) [72]. Importantly, the built and social environments [73] including walking infrastructure was related to more recreational walking habits among community-dwelling older adults just as safety from crime was related to more transport walking [74, 75].

A gait speed of ≤ 0.8 m/s (taking longer than 5 s to walk 4 m), similar to what was observed in our study, has a sensitivity of 0.99 for identifying frailty and implies an increased likelihood that an average participant in this study was frail [76]. In addition, Studenski et al. [77] also demonstrated that every 0.1 m/s reduction in gait velocity in older adults is associated with a 12% increase in mortality. Since the regression line of velocity defines E_1_ on the VFD, E_1_ should relate to frailty and mortality, possibly arising from falls. The inverse association between E_1_ and gait speed among men also suggests that an increase in E_1_ implies an increased tendency to frailty and morbidity. Therefore, as E_1_ increases from zero to 3.5 velots, so also the tendency to fall, frailty and mortality. The vice versa is also true.

Some characteristics (age, BMI, smoking status, alcohol consumption, co-morbidities, and cardiovascular risk factor) associated with falls differed in the population yet the relationship between E_1_ and gait speed was independent of them.Therefore, VFD test reliability is independent of disease/outcome prevalence which explains why the likelihood ratios of the VFD (E_1_) were high, and likewise its reliability, unlike the TUG. The cut-off values for E_1_ quartiles among the males were interpreted as follows: < 1.80 velots signifies inefficient stability region, 1.80–2.51 velots signifies efficient/optimum stability region, 2.52–3.69 velots signifies pre-fall region, and > 3.69 velots signifies falls region; while among the women, the corresponding cut-off values of E_1_ for these regions were: < 1.48 velots, 1.48–2.28 velots, 2.29–3.52 velots and > 3.52velots, respectively. Since men were at a greater risk of falls than women, both sexes should be assessed using separate reference values. From our findings, the optimal categorizations for discrimination between fallers and non-fallers were ≥ 3.78 Velots versus < 3.78 Velots for E_1_ (fallers and non-fallers prevalence 60.71% versus 95.45%, respectively), with a classification accuracy of 0.76. This is better than E_1_ ≥ 3.5 Velots versus < 3.50 Velots versus < 3.50 Velots for E_1_ (fallers and non-fallers prevalence 60.71% versus 95.45%, respectively), as proposed by Eke-Okoro, [26] which has a classification accuracy of 0.56. For the TUG, the optimal categorizations for discrimination between fallers and non-fallers were ≥ 12.81 s versus ≤ 12.81 for TUG times (fallers and non-fallers prevalence 92.86% versus 36.36%, respectively), with a classification accuracy of 0.68, which is better than ≥ 13.50 s versus ≤ 13.50 s, which has a classification accuracy of 0.44.

Though the VFD has not been evaluated in prospective studies, it seems to have a greater fall classification accuracy than the TUG. Our findings regarding the TUG’s sensitivity and specificity were similar to the findings of Barry et al. [1]. Findings from another previous study not only support our findings that the VFD has greater predictive accuracy than TUG (0.61, 95% CI: not given) [78] but also other fall predictive tools such as 25 one-leg stand (0.53, 95% CI: not given) [78], Tinetti balance (0.56, 95% CI: not given) [78], functional reach (0.51, 95% CI: not given) [78], mediolateral sway (0.67, 95% CI: 0.57–0.77) [79] and tandem stand (0.61, 95% CI: 0.49–0.73) [79]. Apart from its specificity and sensitivity values, the overall predictive accuracy of the VFD was similar to the MCHSAT (0.72, 95% CI: 0.63–0.82) (75% specificity, 62% sensitivity) [80]. Since the MCHSAT has a higher specificity than sensitivity, it would discriminate against non-fallers than fallers, similar to TUG, but unlike the VFD.

### Strength and limitations of the study

The strength of this study is that it determined the validity, reliability, and classification accuracy of the VFD in a population of community-dwelling older adults, which has not been done before. To the best of our knowledge, this is the first time that the validity and reliability of the VFD are tested relative to a gold standard (i.e. fallers). For each participant recruited, VFD’s data were collected over a 2 – 3-min time frame, which compared favorably to the 2–5 min required in conducting 3 TUG trials [52, 81] and 45-min time frame recorded for the MCHSAT [80]. Similar to the TUG; the VFD is affordable and maintenance-free, which makes it easily available in resource-poor settings. The study sample was well described, and detailed measurements were conducted while controlling for threats to the internal and external validity. However, the interpretation of the results of this study is limited to the community-dwelling older adult population with similar socio-cultural activities/attributes, and environmental factors as Nigeria/developing countries. This is important because falls are linked to environmental (extrinsic), individual (intrinsic), and activity-related (behavioral) factors [82].

The relatively smaller sample size (*N* = 500) than the projected (*N* = 840) was a limitation to the study but was far greater than nine [26], fifty [45] and sixty-two participants [83] recruited in previous studies which did not report the ROC of the screening tools, unlike our study. Therefore, it is not known whether the sample size had comparable effects on their discriminatory capacities. We measured broad gait parameters to characterize the gait speed of fallers compared to non-fallers which may influence the fall precipitation point (E_1_). The Hanbart stopwatch’s validity is not known but has a resolution of 0.20 s and has been widely used in different research studies. Selection bias is possible as participants were recruited from the health fora and not truly representative of the older adult population in the communities. Nonetheless, some community-based studies on falls [84–87] implemented a similar recruitment approach where it was difficult to enumerate all households with older adults. The convenience sampling technique used in this study is weak in addressing selection bias but is preferable to randomization when selecting individuals with specific attributes (e.g. fallers and non-fallers) in community-based studies. Respondents’ recall bias was possible but was addressed by selecting only participants whose relatives/neighbors corroborated their self-reported fall history. Though the VFD has not been tested across various socio-cultural populations, there is no population for which walking is not a common functional daily activity, unlike other activities, such as deep squatting, utilized by MCHSAT [24, 80].

## Conclusion

The key clinical implication of our findings is that the VFD has a fair discriminatory power and greater accuracy in identifying fallers than the TUG. Consequently, reliance on the TUG as a sole fall discriminatory tool in community-dwelling older adult populations may be misleading and detrimental to health. Therefore, the VFD could replace the TUG as a primary screening tool for the fall status of community-dwelling older adults. However, considering their strengths, a combination of both instruments may be more accurate in fall discrimination and should be explored in future studies.

## Data Availability

The datasets generated and analyzed during the current study are not publicly available due to privacy and ethical concerns but are available from the corresponding author upon reasonable request.
